# Fungistatic Mechanism of Ammonia against Nematode-Trapping Fungus Arthrobotrys oligospora, and Strategy for This Fungus To Survive Ammonia

**DOI:** 10.1128/mSystems.00879-21

**Published:** 2021-09-14

**Authors:** Tong Liu, Xi Long, Jia-Peng Zhou, Dong-Wei Tian, Yun-He Yang, Cheng-Gang Zou, Jian-Ping Xu, Ming-He Mo, Ke-Qin Zhang

**Affiliations:** a State Key Laboratory for Conservation and Utilization of Bio-Resources in Yunnan, Yunnan University, Kunming, People’s Republic of China; b Key Laboratory of Microbial Diversity in Southwest China, Yunnan University, Kunming, People’s Republic of China; c Department of Biology, McMaster University, Ontario, Canada; d Engineering Research Center of Biocontrol of Plant Disease & Pest, Yunnan University, Kunming, People’s Republic of China; Oak Ridge National Laboratory

**Keywords:** soil fungistasis, fungistatic mechanism, multi-omics, ER stress, *Arthrobotrys oligospora*

## Abstract

Soil fungistasis is a phenomenon in which the germination and growth of fungal propagules is widely inhibited in soils. Although fungistatic compounds are known to play important roles in the formation of soil fungistasis, how such compounds act on soil fungi is little studied. In this study, it was found that ammonia (NH_3_) induced global protein misfolding marked by increased ubiquitination levels of proteins (ubiquitylome data and Western blot verification). The misfolded proteins should trigger the endoplasmic reticulum (ER) stress, which was indicated by electron microscope image and proteome data. Results from the mutants of BiP and proteasome subunit alpha 7 suggested that ER stress played a mechanistic role in inhibiting conidial germination. Results from proteome data indicated that, to survive ammonia fungistasis, conidia first activated the unfolded protein response (UPR) to decrease ER stress and restore ER protein homeostasis, and the function of UPR in surviving ammonia was confirmed by using mutant strains. Second, ammonia toxicity could be reduced by upregulating carbon metabolism-related proteins, which benefited ammonia fixation. The results that metabolites (especially glutamate) could relieve the ammonia fungistasis confirmed this indirectly. Finally, results from gene knockout mutants also suggested that the fungistatic mechanism of ammonia is common for soil fungistasis. This study increased our knowledge regarding the mechanism of soil fungistasis and provided potential new strategies for manipulating soil fungistasis.

**IMPORTANCE** Soil fungistasis is a phenomenon in which the germination and growth of fungal propagules is widely inhibited in soil. Although fungistatic compounds are known to play important roles in the formation of soil fungistasis, how such compounds act on soil fungi remains little studied. This study revealed an endoplasmic reticulum stress-related fungistatic mechanism with which ammonia acts on Arthrobotrys oligospora and a survival strategy of conidia under ammonia inhibition. Our study provides the first mechanistic explanation of how ammonia impacts fungal spore germination, and the mechanism may be common for soil fungistasis. This study increases our knowledge regarding the mechanism of soil fungistasis in fungal spores and provides potential new strategies for manipulating soil fungistasis.

## INTRODUCTION

Plant-parasitic nematodes are responsible for global agricultural losses of approximately $100 billion annually (1). More than 4,100 species of plant-parasitic nematodes have been documented to date (2), and root-knot nematodes (RKNs) (*Meloidogyne* spp.) are of major worldwide economic importance (3). RKNs damage the plant root and repress the uptake of water and nutrients, and they also predispose plants to be attacked by other pathogens through mechanical damage (4). Chemical nematicides are the most reliable resources to control RKNs; however, they are increasingly being withdrawn due to their toxicity to humans and the environment (5). Moreover, the overuse of chemical nematicides has caused drug resistance (6–8). Alternative methods, including the application of fungal biocontrol agents, are needed to be developed to control these pests.

Nematophagous fungi are natural enemies of nematodes and comprise four main groups of fungi—nematode-trapping fungi, endoparasitic fungi, opportunistic fungi, and toxic fungi (9, 10). Nematophagous fungi, including Arthrobotrys oligospora, Paecilomyces lilacinus, Verticillium chlamydosporium, and Pochonia chlamydosporia, are among those most used for the biological control of RKNs (11, 12). For the successful control of RKNs, a sufficient population density of fungal agents in the rhizosphere is needed. However, soil fungistasis (mycostasis) strongly represses the germination and growth of fungal biocontrol agents (13), rendering them inefficient in soils.

Soil fungistasis is a phenomenon in which most natural soils suppress the germination and growth of fungi to various degrees (14), which was first described by Dobbs and Hinson (15). The intensity of fungistasis depends on the physical and chemical properties of the soil and soil microbial activity (14, 16–18). The soil microbial community has been demonstrated to play an important role in soil fungistasis (19–21). Microbial community carbon competition is one of the causes of fungistasis (21, 22). Another reason is that antifungal compounds of microbiological origin inhibit conidial germination and hyphal growth (23, 24). The importance of volatiles as major fungistatic factors has long been recognized (25–27). Many volatiles produced by soil microorganisms have been identified and shown to reduce or inhibit conidial germination and/or hyphal growth in various fungi (24, 28, 29). Based on these studies, Garbeva et al. reviewed the basic characteristics of soil fungistasis (30), including the effects of soil physical and chemical properties on the fungistatic intensity, and the relationship between fungistasis, soil microorganisms, and their compounds. However, the molecular mechanisms by which fungistatic compounds suppress the germination and growth of fungi have not been elucidated. Understanding the fungistatic mechanism is important for ensuring the efficacy of biocontrol agents against RKNs in soil environments.

Ammonia (NH_3_) is the first identified fungistatic compound found in alkaline soil (26). Degradation of natural protein-rich resources (e.g., carcasses, whey, manure, and compost) is accompanied by bacterial ammonia emission (31). In addition, ammonia released from nitrogenous fertilizers has been shown to inhibit the growth of Aspergillus niger (32) and the conidial germination of Penicillium griseofulvum and Fusarium graminearum (33). Furthermore, urease-producing bacteria can catalyze the conversion of urea to ammonia (34), and urea, as a nitrogenous fertilizer, is often applied near the crop root in agriculture. Moreover, the production of ammonia by *Streptomyces* species has been shown as a low-cost and long-distance antibiotic strategy (35), and *Streptomyces* is one of the dominant bacterial genera in soils (36–38). Together, these results suggest that ammonia is a key rhizosphere fungistatic factor that represses the conidial germination of nematophagous fungi.

The nematode-trapping fungus *Arthrobotrys oligospora* ATCC 24927 is a biocontrol fungus used to control RKNs. Based on the proteome data, our previous study revealed that the conidial germination of *A. oligospora* ATCC 24927 was inhibited by ammonia, and repression of protein synthesis was likely a reason for the inhibition of conidia germination (39). To reveal the detailed fungistatic mechanism of ammonia and the survival strategy of conidia under ammonia fungistasis, we further analyzed the transcriptome and ubiquitylome of conidia under ammonia fungistasis and compared them to those of fresh conidia in the absence of ammonia. Based on the combined analysis of these multi-omics data and results from mutant strains, the fungistatic mechanism of ammonia against *A. oligospora* and the strategy for this fungus to survive ammonia is revealed in this study.

## RESULTS AND DISCUSSION

### The fungistatic role of ammonia (NH_3_) cannot be attributed to alkaline pH or NH_4_^+^ ions.

**FIG 1:**
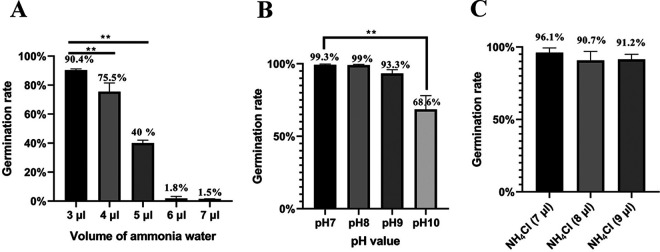
Conidial germination rates at 24 h under different fungistatic conditions. (A) The conidial germination rate was tested under the fungistatic stress of ammonia water. (B) The conidial germination rate was tested under the fungistatic stress of alkaline pH. (C) The conidial germination rate was tested under the fungistatic stress of NH_4_Cl. NH_4_Cl (7 μl), NH_4_Cl (8 μl), and NH_4_Cl (9 μl) mean that the molar amounts of NH_4_Cl were same as those of NH_3_ in 7, 8, and 9 μl of ammonia water, respectively. **, *P* < 0.01.

Ammonia is a key rhizosphere fungistatic factor in agriculture soils. It can inhibit the conidial germination of the nematophagous fungus *A. oligospora*. As shown in Fig. 1A, the conidial germination rate on water agar (WA) medium decreased with increasing volume of ammonia water. Nearly complete inhibition was observed when 6 μl of ammonia water was used. The inhibitory dose of ammonia on the WA medium was slightly lower than that on the corn meal agar (CMA) medium (39).

As we know, a small portion of ammonia can ionize as NH_4_^+^ and OH^–^ in water. To investigate which ion mediates the fungistatic role of ammonia, the effects of alkaline pH and NH_4_^+^ ions on conidial germination were assessed. When the pH reached 10, significant inhibition of conidial germination by alkaline pH was observed (Fig. 1B). However, the addition of as many as 9 μl of ammonia water to 5 ml of deionized water just produced a pH of 8. The same molar amount of NH_4_Cl as that of NH_3_ in 7, 8, and 9 μl of ammonia water inhibited conidial germination very slightly (Fig. 1C). These results suggested that the fungistatic role of ammonia cannot be attributed to alkaline pH or NH_4_^+^ ions, which should be attributed to the direct role of NH_3_.

### Fungistatic stress of ammonia resulted in global protein misfolding.

**FIG 2:**
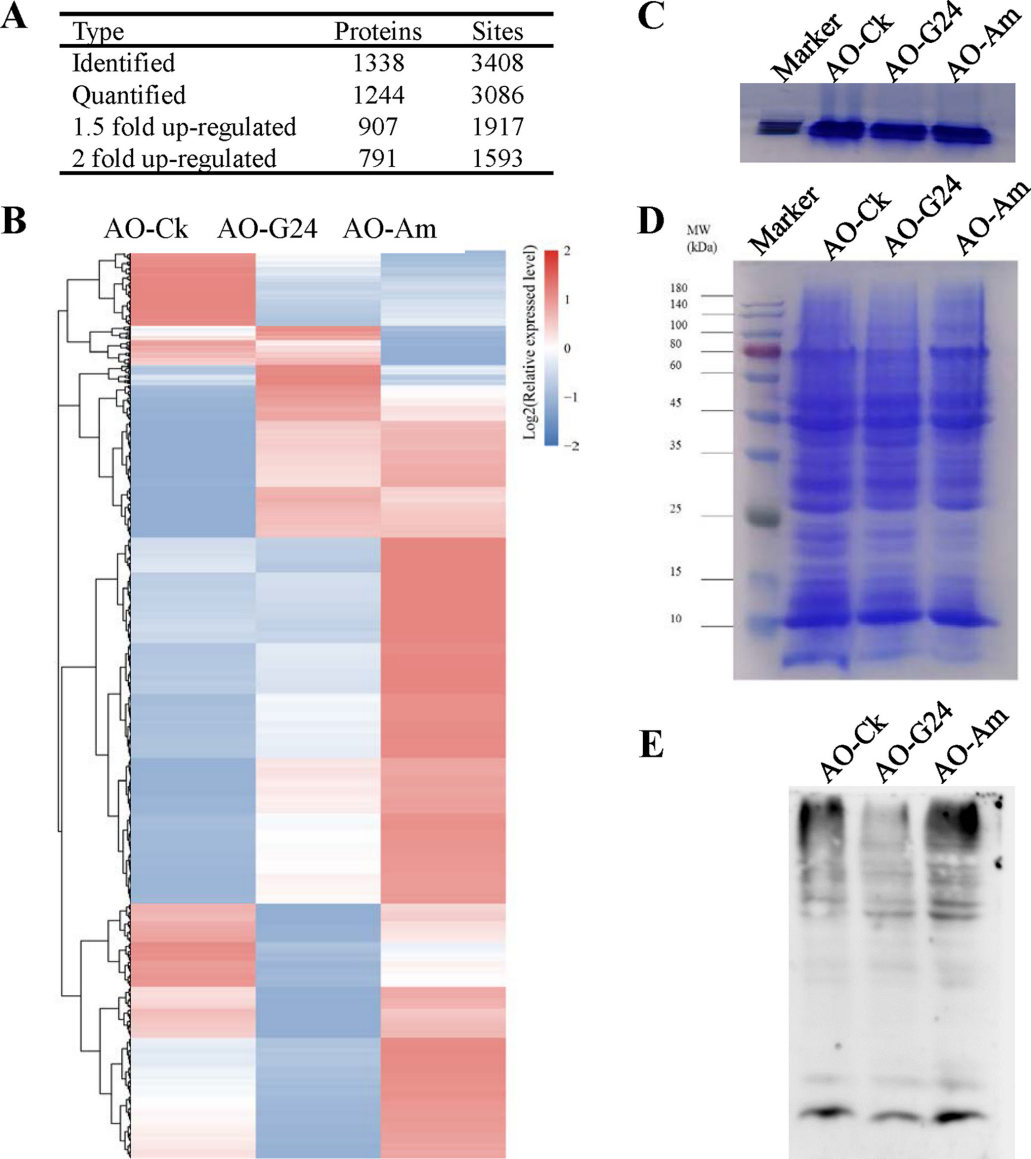
Detection of ubiquitination by ubiquitylome analysis and Western blot analysis. (A) Statistical result of ubiquitylated proteins and sites in ubiquitylome data. (B) Heat map analysis of quantified ubiquitylation sites. (C) Equalization of total amounts of proteins between different conidium sample by running short SDS-PAGE (0.3 cm). (D) SDS-PAGE analysis of total proteins. (E) Detection of ubiquitination by anti-ubiquitin antibody in Western blot. AO-Ck, fresh conidia; AO-G24, germinated conidia; AO-Am, fungistatic conidia.

Ubiquitylome analysis revealed that global protein misfolding in Asian rice increased significantly under thermal stress (40). The fungistatic stress of ammonia can also induce protein misfolding. In this study, the ubiquitylomes of fresh, germinated, and fungistatic conidia were determined and compared. In total, 3,408 ubiquitylated lysine sites of 1,338 proteins were identified, and 3,086 sites of 1,244 proteins were quantified (Fig. 2A). When fresh conidia were used as the control, the ubiquitylation levels of 1,917 sites in 907 proteins increased by at least 1.5-fold in fungistatic conidia. Among these proteins, ubiquitylation levels of 1,593 sites in 791 proteins increased 2-fold or more. The heat map of quantified ubiquitylation sites also showed that the ubiquitylation profile of fungistatic conidia was very different from that of fresh conidia and germinated conidia, and ubiquitylation levels of many proteins increased markedly in fungistatic conidia (Fig. 2B). The total amounts of proteins were equalized by running short SDS-PAGE (0.3 cm) and quantifying the gray value (Fig. 2C), and then the same amounts of proteins from the three conidium samples was used for SDS-PAGE and Western blotting (Fig. 2D and E). The results confirmed that ubiquitylation levels of proteins in fungistatic conidia increased obviously.

Protein ubiquitination is known to play important regulatory roles in eukaryotic cells, such as cell division, fate specification, and migration (41). In addition, ubiquitylation is the hallmark of misfolded protein, and ubiquitylated misfolded proteins within the endoplasmic reticulum are transported to the 26S proteasome for degradation (42). The globally increased ubiquitylation levels of proteins in fungistatic conidia might suggest a global protein misfolding induced by the fungistatic stress of ammonia.

### Misfolded proteins triggered endoplasmic reticulum (ER) stress.

**FIG 3:**
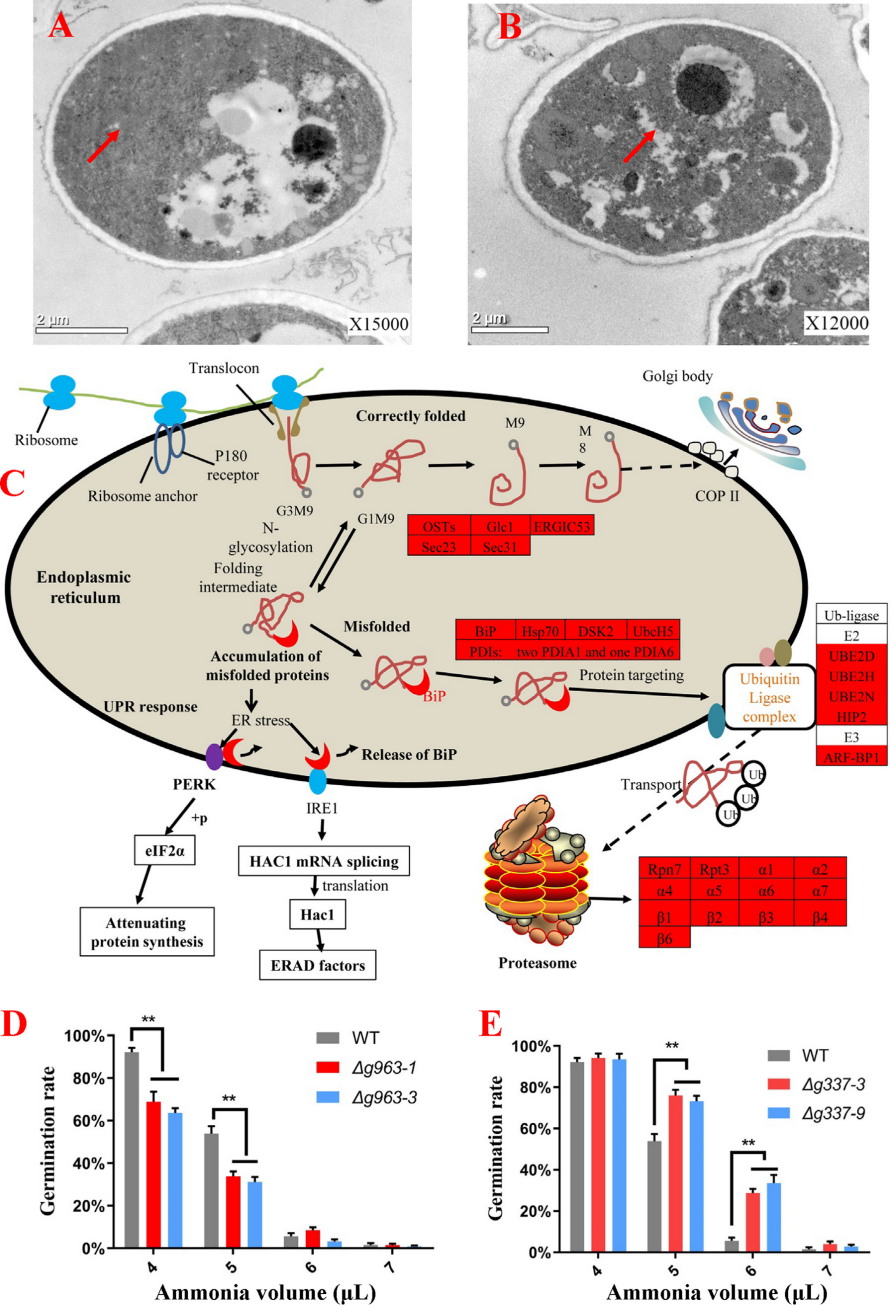
Analyses of ER stress induced by ammonia fungistasis. (A and B) Ultrastructural analyses of fresh conidia (A) and fungistatic conidia (B) by transmission electron microscopy; the arrows indicate the possible area of ER. (C) Distribution of up-expressed proteins in “proteasome,” “protein processing in endoplasmic reticulum,” and “ubiquitin ligase complex”; up-expressed proteins are shown with a red background. (D) Germination rate testing of gene *AOL_s00054g963* knockout mutants. (E) Germination rate testing of gene *AOL_s00210g337* knockout mutants. WT, wild-type strain. Δ*g963-1* and Δ*g963-3*, knockout mutants of gene *AOL_s00054g963*. Δ*g337-3* and Δ*g337-9*, knockout mutants of gene *AOL_s00210g337*. **, *P* < 0.01.

It was reported that the accumulation of misfolded proteins could trigger ER stress (43). As shown in Fig. 3A, the ER was continuous in the fresh conidia but fragmented in the ammonia-fungistatic conidia (Fig. 3B). The proteome of ammonia-fungistatic conidia reported by our lab (39) offered more evidence for ammonia-induced ER stress. As a response to the stress of ammonia, 392 proteins were upregulated, and a KEGG pathway analysis of these proteins was performed (Table 1). The result showed that 13, 12, and 5 up-expressed proteins are involved in “proteasome,” “protein processing in endoplasmic reticulum,” and “ubiquitin mediated proteolysis,” respectively. These proteins are involved in protein processing of correctly folded proteins and proteolysis of misfolded protein (Fig. 3), and upregulation of these proteins is a hallmark of ER stress. As shown in Fig. 3C, misfolded proteins in ER are bound by the BiP protein and transported to the ubiquitin modification system. After ubiquitylation, the misfolded proteins are transferred to the proteasome for proteolysis. Competitive binding of BiP by misfolded proteins can result in the release of BiP from PERK and IRE1 and stimulate the unfolded protein response (UPR) (44), which includes three branches in mammalian cells and two branches in the *A. oligospora*. Stimulation of the IRE1 branch increases the expression of the ubiquitin-proteasome system. Thus, up-expression of these proteins, especially the overall up-expression of BiP and proteasome subunit proteins, suggested that ammonia induced ER stress.

**TABLE 1:** KEGG pathway analyses of up-expressed proteins in ammonia fungistatic conidia

KEGG pathway	No. of proteins
Purine metabolism	17
Cysteine and methionine metabolism	15
Proteasome	13
Protein processing in endoplasmic reticulum	12
2-Oxocarboxylic acid metabolism	12
Glyoxylate and dicarboxylate metabolism	10
Endocytosis	9
Glycine, serine, and threonine metabolism	9
Pyruvate metabolism	9
Cell cycle—yeast	9
Glycolysis	8
Oxidative phosphorylation	8
Pyrimidine metabolism	8
Glutathione metabolism	8
Peroxisome	8
Meiosis—yeast	7
Thermogenesis	7
Citrate cycle	7
Valine, leucine, and isoleucine biosynthesis	7
Sulfur metabolism	6
RNA degradation	6
Spliceosome	6
Alanine, aspartate, and glutamate metabolism	6
Ribosome biogenesis in eukaryotes	5
Ubiquitin-mediated proteolysis	5
RNA transport	5
Ribosome	5

The BiP and proteasome were upregulated in ammonia fungistatic conidia, and their function in resisting ammonia fungistasis was further studied. The genes *AOL_s00054g963* and *AOL_s00210g337*, encoding the proteasome subunit α7 and protein BiP (Table S1), respectively, were knocked out (Fig. S1), and knockout mutants were obtained. The function of BiP and proteasome subunit α7 was assessed by detecting the conidial germination rate of mutants under the fungistatic stress of ammonia. The deletion of these two genes had no evident impact on conidial germination rates under normal conditions (without ammonia). However, as shown in Fig. 3D, the conidial germination rates of α7 mutants Δ*g963-1* and Δ*g963-3* were significantly lower than that of the wild-type strain when the fungistatic stress of ammonia was given. In contrast, the BiP deletion mutants Δ*g337-3* and Δ*g337-9* showed opposite results; they had significantly higher conidial germination rates than the wild-type strain (Fig. 3E). This could be due to the stimulation of UPR by BiP deletion (45). The UPR can decrease the load of misfolded proteins by attenuating protein translation, increasing the folding capacity of ER by upregulating the chaperone proteins (Table S2), and increasing ER-associated degradation (ERAD) to degrade unfolded proteins at the proteasome (46, 47). The results from these mutants suggest that ER stress induced by ammonia is an important mechanistic reason for inhibiting conidial germination, and UPR is important for *A. oligospora* to resist the fungistatic stress of ammonia.

10.1128/mSystems.00879-21.1 TABLE S1 BLAST searches of the human BiP protein (GenBank accession no. CAA61201.1) and the yeast BiP protein Kar2p (GenBank accession no. AJV43944.1) (Eukaryot Cell. 2005 4:2008–2016. doi:10.1128/EC.4.12.2008-2016.2005) were conducted against the protein data base of *Arthrobotrys oligospora* ATCC 24927. Protein encoded by *AOL_s00210g337* has the highest identity in both BLAST tests, and it contains an HSPA5-like nucleotide-binding domain which is contained in BiP/GRP78 protein. Protein encoded by *AOL_s00215g255* also has high identity, but it contains an HSPA1-2_6-8-like nucleotide-binding domain. Based on the BLAST results, protein encoded by *AOL_s00210g337* is annotated as BiP protein. Download Table S1, DOCX file, 0.01 MB. Copyright © 2021 Liu et al. 2021 Liu et al. https://creativecommons.org/licenses/by/4.0/ This content is distributed under the terms of the Creative Commons Attribution 4.0 International license.

10.1128/mSystems.00879-21.2 TABLE S2 Molecular chaperone up-regulated proteins in fungistatic conidia Table S2, DOCX file, 0.02 MB. Copyright © 2021 Liu et al. 2021 Liu et al. https://creativecommons.org/licenses/by/4.0/ This content is distributed under the terms of the Creative Commons Attribution 4.0 International license.

10.1128/mSystems.00879-21.4 FIG S1 Identification of double crossover homologous recombinants by PCR. The hygromycin-resistant transformants were selected and identified by PCR, two double crossover homologous recombinants (1-3 and 3-2 in panel A) for gene *AOL_s00054g963* were found. The subcultures were identified a second time for confirmation of knockout mutants of genes *AOL_s00054g963* (B); strains 1-3 and 3-2 for gene *AOL_s00054g963* are genetically stable transformants, and are named Δ*g963-1* and Δ*g963-3*, respectively. Two double crossover homologous recombinants (3-4 and 9-1 in panel C) for gene *AOL_s00210g337* were found. The subcultures were identified a second time for confirmation of knockout mutants of genes *AOL_s00210g337* (D). Strains 3-4 and 9-1 for gene *AOL_s00210g337* are genetically stable transformants and are named Δ*g337-3* and Δ*g337-9*, respectively. Download FIG S1, JPG file, 0.4 MB. Copyright © 2021 Liu et al. 2021 Liu et al. https://creativecommons.org/licenses/by/4.0/ This content is distributed under the terms of the Creative Commons Attribution 4.0 International license.

### Protein misfolding was correlated with protein downregulation in fungistatic conidia.

**FIG 4:**
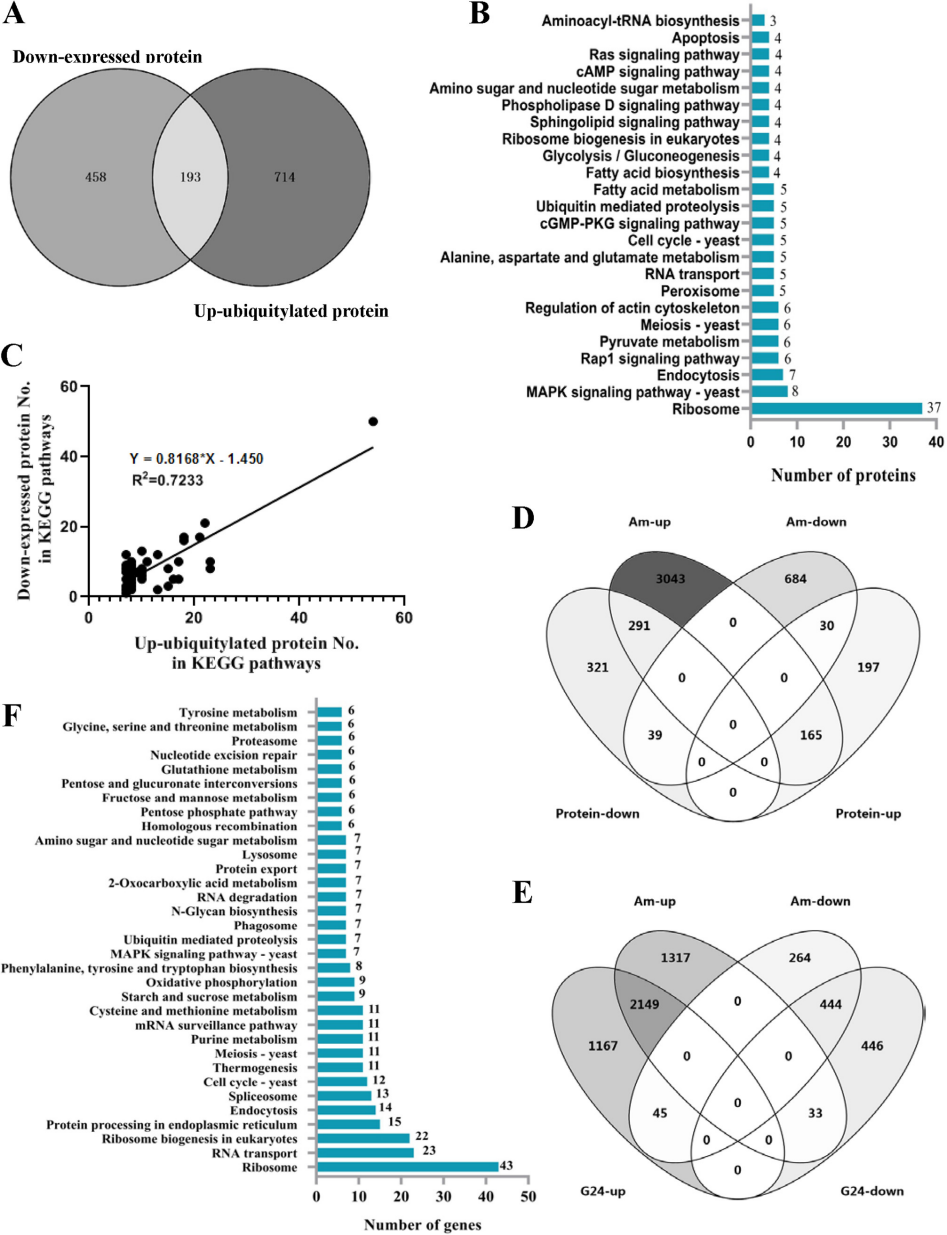
Correlation analysis between up-ubiquitylated proteins, up-transcribed genes, and down-expressed proteins. (A) Venn diagram analysis of up-ubiquitylated proteins and down-expressed proteins. (B) KEGG pathway analysis of common proteins between up-ubiquitylated proteins and down-expressed proteins. (C) Linear regression analysis of KEGG pathway distribution between up-ubiquitylated proteins and down-expressed proteins. (D) Venn diagram analysis of differentially transcribed genes and differentially regulated proteins in fungistatic conidia. (E) Venn diagram analysis of differentially transcribed genes in germinated conidia (G24) and fungistatic conidia (Am). (F) KEGG pathway analyses of 1,350 up-transcribed genes in fungistatic conidia.

Under ammonia treatment, except for 392 up-expressed proteins, 651 proteins were down-expressed in the proteome of fungistatic conidia. The Venn diagram showed that 193 up-ubiquitylated proteins could be matched to the downregulated proteins (Fig. 4A). These 193 proteins were further mapped to KEGG pathways (Fig. 4B), and the most outstanding characteristic is that 37 proteins are ribosome proteins. KEGG pathway analyses of 907 up-ubiquitylated proteins and 651 down-expressed proteins in fungistatic conidia were performed and compared (Table 2). As shown in Table 2, the distribution of up-ubiquitylated proteins in the KEGG pathways was similar to that of down-expressed proteins. Using the data in Table 2 for linear regression analysis, the results showed a correlation in pathway distribution between up-ubiquitylated proteins and down-expressed proteins (Fig. 4C). These results showed that protein misfolding, marked by up-ubiquitylation, contributed to protein downregulation in fungistatic conidia.

**TABLE 2:** Comparison of KEGG pathways containing up-ubiquitylated proteins or down-expressed proteins

KEGG pathways	No. of up-ubiquitylated proteins	No. of down-expressed proteins
Ribosome	54	50
Protein processing in endoplasmic reticulum	23	8
Endocytosis	23	10
RNA transport	22	21
MAPK signaling pathway—yeast	21	17
Cell cycle—yeast	18	17
Meiosis—yeast	18	16
Proteasome	17	5
Autophagy—yeast	17	10
Glycolysis/gluconeogenesis	16	5
Thermogenesis	15	8
Oxidative phosphorylation	15	3
Cysteine and methionine metabolism	13	2
Ubiquitin-mediated proteolysis	13	12
Alanine, aspartate, and glutamate metabolism	11	10
Mitophagy—yeast	10	8
Amino sugar and nucleotide sugar metabolism	10	6
2-Oxocarboxylic acid metabolism	10	6
Pyruvate metabolism	10	7
Peroxisome	10	5
Ribosome biogenesis in eukaryotes	10	13
Purine metabolism	10	7
Starch and sucrose metabolism	10	6
Regulation of actin cytoskeleton	9	7
Glyoxylate and dicarboxylate metabolism	8	2
RNA degradation	8	9
Ras signaling pathway	8	7
Sphingolipid signaling pathway	8	7
Pentose phosphate pathway	8	3
AMPK signaling pathway	8	5
mRNA surveillance pathway	8	10
Phagosome	8	4
cGMP-PKG signaling pathway	8	5
PI3K-Akt signaling pathway	8	8
Lysosome	7	5
Aminoacyl-tRNA biosynthesis	7	12
Rap1 signaling pathway	7	7
Arginine and proline metabolism	7	3
Arginine biosynthesis	7	5
Fructose and mannose metabolism	7	2
Calcium signaling pathway	7	3
Spliceosome	7	9
mTOR signaling pathway	7	8
Glutathione metabolism	7	3
Phospholipase D signaling pathway	7	7
Citrate cycle (TCA cycle)	7	2
Galactose metabolism	7	1

Generally speaking, protein expression levels depend on not only translation efficiency but also on transcription activities. In our previous study (39), it was found using reverse transcription PCR (RT-PCR) that, between 26 tested genes, transcription levels of 23 genes are higher or not less than those of fresh conidia and germinated conidia. To investigate whether the gene transcription of 651 down-expressed proteins was inhibited under the fungistatic stress of ammonia, the 651 down-expressed proteins were matched to differentially transcribed genes in fungistatic conidia. The result showed that there were only 39 down-expressed proteins (6%), of which transcription levels were downregulated in fungistatic conidia (Fig. 4D). This meant that the transcription of most genes encoding down-expressed proteins was not inhibited by ammonia, and this was consistent with the result of our previous study. Therefore, protein down-expression could not be attributed to inhibition of gene transcription.

In addition, compared to the transcriptome of germinated conidia, except for 2,149 common genes up-transcribed in both germinated and fungistatic conidia, 1,350 genes were up-transcribed just in fungistatic conidia (Fig. 4E), and these genes were mapped to KEGG pathways (Fig. 4F). More than 100 genes were involved in pathways related to protein synthesis, especially ribosome, RNA transport, and ribosome biogenesis (Table S2). A correlation in pathway distribution between down-expressed proteins and up-transcribed genes was also found (Table S3 and Fig. S2). This meant that, as a response to protein down-expression, the fungistatic conidia gave a precise response at the transcription level—gene transcription levels of most down-expressed proteins were upregulated. However, the upregulation of gene transcription might not be able to rescue protein down-expression because UPR only allowed selective translation of specific mRNAs while repressing the translation of most mRNAs to maintain ER protein homeostasis (48).

10.1128/mSystems.00879-21.3 TABLE S3 KEGG pathway types of 651 down-expressed proteins and 1,350 up-transcribed genes were compared. Download Table S3, DOCX file, 0.02 MB. Copyright © 2021 Liu et al. 2021 Liu et al. https://creativecommons.org/licenses/by/4.0/ This content is distributed under the terms of the Creative Commons Attribution 4.0 International license.

10.1128/mSystems.00879-21.5 FIG S2 Correlation analysis in pathway distribution between down-expressed proteins and up-transcribed genes. Download FIG S2, JPG file, 0.07 MB. Copyright © 2021 Liu et al. 2021 Liu et al. https://creativecommons.org/licenses/by/4.0/ This content is distributed under the terms of the Creative Commons Attribution 4.0 International license.

### Survival strategy of conidia under ammonia fungistasis.

**FIG 5:**
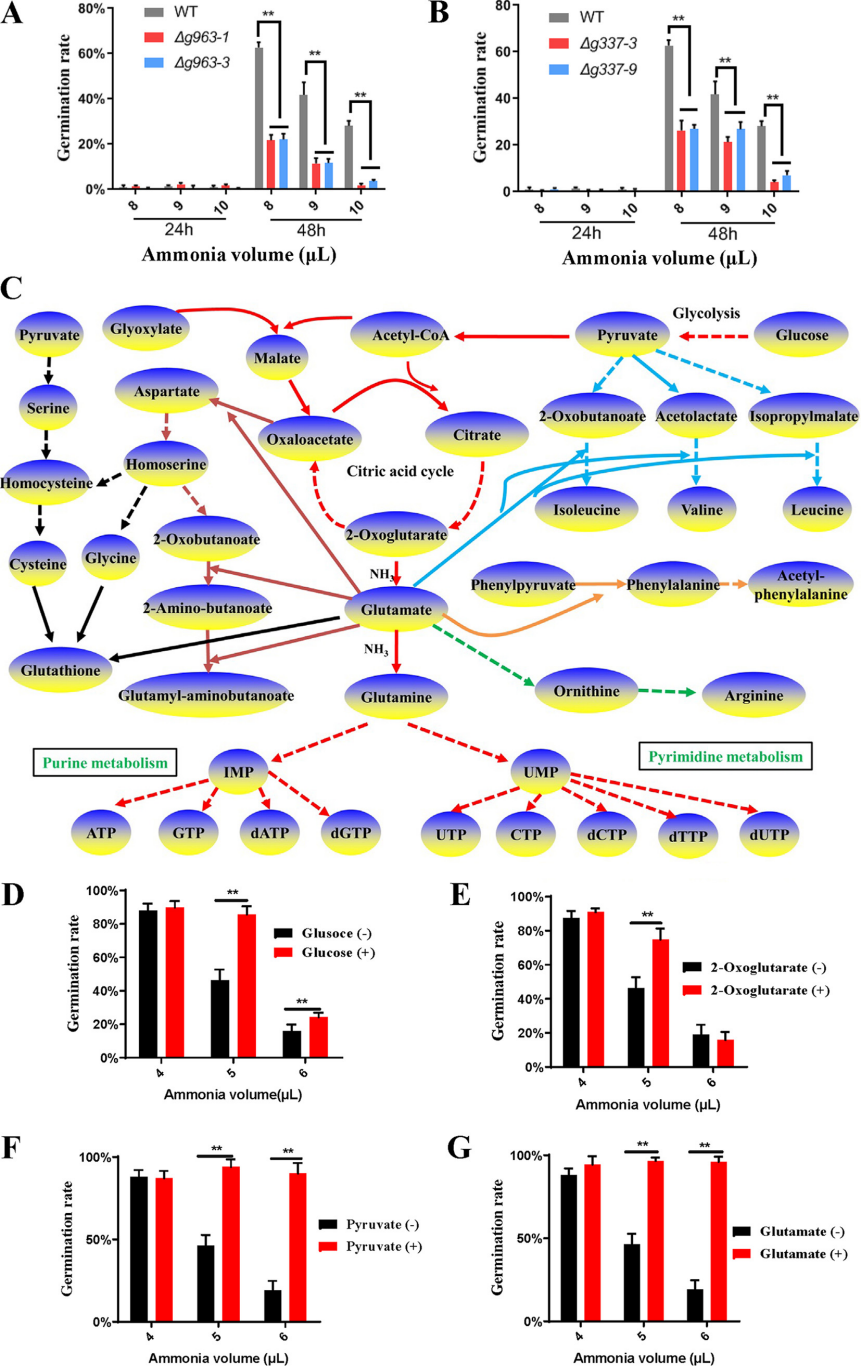
Survival strategy analysis of conidia under ammonia fungistasis. (A) Survival ability test of proteasome α7 mutants under ammonia fungistasis. WT, wild-type strain. Δ*g963-1* and Δ*g963-3*, knockout mutants of gene *AOL_s00054g963*. (B) Survival ability test of BiP mutants under ammonia fungistasis. Δ*g337-3* and Δ*g337-9*, knockout mutants of gene *AOL_s00210g337*. (C) Ammonia fixation pathways in fungistatic conidia. The red lines represent the glycolysis, citric acid cycle, glutamate synthesis, purine metabolism, and pyrimidine metabolism. The blue lines represent the valine, leucine, and isoleucine biosynthesis. The brown lines represent the aspartate biosynthesis and metabolism. The black lines represent serine, cysteine, and glycine metabolism and glutathione synthesis. The orange line represents phenylalanine synthesis. The green line represents ornithine and arginine synthesis. Dotted lines mean multistep reactions, and solid lines mean one-step reactions. (D to G) Test of the conidial germination rates of the wild-type strain on WA medium containing glucose, 2-oxoglutarate, pyruvate, and glutamate under the ammonia fungistasis. +, with compound; –, without compound. **, *P* < 0.01.

We previously reported that the fungistatic conidia could resume germination after removal of ammonia (39). This suggested that the conidia could survive the ammonia fungistasis. As shown in Fig. 3C, UPR should play an important role in the survival of the ammonia-induced fungistasis. First, UPR inhibited global protein translation and decreased the load of misfolded proteins to relieve ER stress. Second, UPR allowed the selective translation of specific mRNAs to upregulate some proteins. For example, UPR upregulated the ubiquitin-proteasome system to promote the degradation of misfolded proteins and upregulated molecular chaperones (Table S2) to boost the folding capacity of the ER (49). The results from the mutant strains confirmed this indirectly. The conidial germination of both the wild-type strain and proteasome α7 mutants (Δ*g963-1* and Δ*g963-3*) was almost completely inhibited by 8, 9, and 10 μl of ammonia water, with the germination rate at 24 h being near 0; 24 h after the removal of ammonia, compared to the wild-type strain, many fewer mutant conidia resumed germination (Fig. 5A). Similar results were observed in the BiP deletion mutants (Δ*g337-3* and Δ*g337-9*) (Fig. 5B). These results confirmed that UPR is very important for *A. oligospora* to survive ammonia fungistasis.

In addition, upregulation of metabolism-related proteins seemed to benefit conidial survival. A total of 116 up-expressed proteins were involved in the metabolisms of purine, cysteine/methionine, 2-oxocarboxylic acid, glyoxylate/dicarboxylate, glycine/serine/threonine, pyruvate, glycolysis, pyrimidine, glutathione, citrate cycle, valine/leucine/isoleucine, and alanine/aspartate/glutamate (Table 2). By checking the roles of these proteins in these pathways, we found that the upregulation of these proteins was beneficial for ammonia fixation (Fig. 5C). For example, glycolysis, citrate cycle, glyoxylate metabolism, and pyruvate metabolism can provide precursor carbon skeletons for amino acid biosynthesis, including oxaloacetate, 2-oxoglutarate, 2-oxobutanoate, acetolactate, and isopropylmalate. Among these, 2-oxoglutarate is directly used to fix ammonia and produce glutamate. Glutamate provides the amino group for oxaloacetate, 2-oxobutanoate, acetolactate, and isopropylmalate to produce aspartate, isoleucine, valine, and leucine, respectively (indicated by the blue arrows). 2-Oxobutanoate also reacts with 2 glutamate and produces glutamyl-aminobutanoate (indicated by a brown arrow). Glutamate can also provide amino groups for the biosynthesis of other amino acids, including phenylalanine and arginine (indicated by the orange and green arrows). In addition, the up-expressed proteins in cysteine, glycine, and serine metabolism can promote the synthesis of cysteine and glycine, which can be used to synthesizes glutathione (indicated by the black arrow). Moreover, glutamate can fix ammonia directly and produce glutamine, which is involved in the synthesis of purine and pyrimidine (indicated by the red arrows). Upregulation of these proteins can promote ammonia fixation, thereby reducing the toxicity of ammonia to conidia. Further results showed that glucose, 2-oxoglutarate, pyruvate, and glutamate (Fig. 5D to G) could relieve the ammonia inhibition and improve the conidial germination rate; pyruvate and glutamate had the best relief effect. These results proved indirectly that upregulation of metabolism-related proteins could promote the fixation of ammonia and should be beneficial for conidia to survive ammonia fungistasis, and glutamate played a central role in these processes.

### The mechanism of ammonia fungistasis is common for soil.

**FIG 6:**
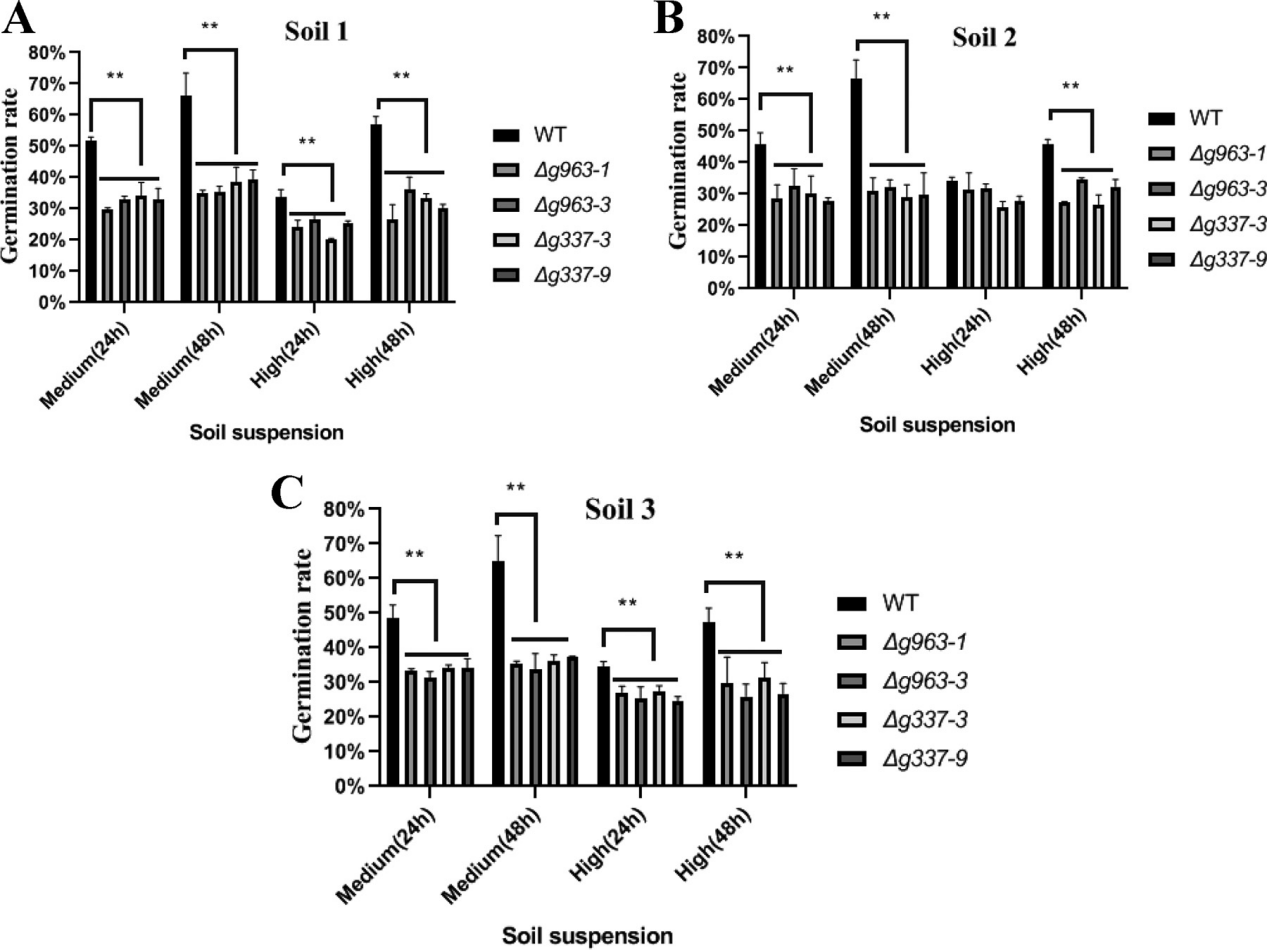
Conidial germination analysis of conidia under soil fungistasis from different soil samples (A to C). WT, wild-type strain. Δ*g963-1* and Δ*g963-3*, knockout mutants of gene *AOL_s00054g963* encoding proteasome α7 subunit. Δ*g337-3* and Δ*g337-9*, knockout mutants of gene *AOL_s00210g337* encoding BiP protein. Medium (24 h), the conidia were cultured in medium-fungistatic soil suspension for 24 h. Medium (48 h), the conidia were cultured in medium-fungistatic soil suspension for 24 h, moved to deionized water, and placed there for 24 h. High (24 h), the conidia were cultured in high-fungistatic soil suspension for 24 h. High (48 h), the conidia were cultured in high-fungistatic soil suspension for 24 h, moved to deionized water, and placed there for 24 h. **, *P* < 0.01.

The mechanism of ammonia fungistasis may be common for soil. The results from the mutant strains confirmed this. The conidial germination rates of mutant strains at 24 h (proteasome α7 mutants and BiP deletion mutants) in the medium and high fungistatic soil suspensions were significantly lower than those of the wild-type strain (Fig. 6A). Resumed germination was observed in the wild-type strain 24 h after the removal of conidia from soil suspensions to deionized water; however, few mutant conidia resumed germination. Similar results were obtained in the test using other soil samples (Fig. 6B and C). These results suggested that the mechanism of ammonia fungistasis could be applied to soils. However, the soils contain many fungistatic compounds; other fungistasis mechanisms may well exist in soils.

In conclusion, this study increased our knowledge regarding the mechanism of soil fungistasis against fungal spores. Based on research results from the past 7 decades, the formation of soil fungistasis can be attributed at least partially to physicochemical properties (pH, heavy metal ions, moisture, and oxygen content) and nutrient deficiency in soil. It is well recognized that the fungistatic microbial communities (diversity and interactions) and the fungistatic compounds produced by soil microorganisms play important roles in generating and maintaining soil fungistasis (30). Moreover, the ecological significance of soil fungistasis has been recognized (35, 50). Soil fungistasis is a result of soil microorganisms competing for ecological niches and nutrients in soil. However, there is no research on how the molecular mechanism of soil fungistasis inhibits the spore germination of fungi. This study provided the first insight into this issue and will promote the transformation of research attention from the cause of formation to the molecular mechanism, which is of great significance.

It was reported that ammonia (NH_4_Cl) could induce oxidative stress, ER stress, and/or cell apoptosis in different tissues or organisms (51, 52). However, we found that NH_4_Cl had little inhibitory effect on conidial germination (Fig. 1C). Thus, the role of NH_3_ should be different from that of NH_4_Cl. Our results showed that NH_3_ induced protein misfolding, which thereby induced ER stress; in order to provide more evidence for this conclusion, subcellular localization analysis of the up-ubiquitinated proteins was performed. The result showed that these proteins located mainly in cytoplasm (36.2%), nucleus (24.6%), mitochondria (16.6%), and plasma membrane (15.8%) (Fig. S3). It meant that besides the ER-processed secretory proteins, misfolding of cytoplasm proteins also increased, and the NH_3_-induced protein misfolding was global. If the increased protein misfolding is a result of ER stress, the up-ubiquitinated proteins should be enriched in ER-processed secretory proteins. In contrast, the subcellular localization result suggested that increased protein misfolding is not a result but a cause of ER stress. The accumulation of misfolded proteins in ER should trigger the ER stress.

10.1128/mSystems.00879-21.6 FIG S3 Subcellular localization analysis of up-ubiquitinated proteins. We used WoLF PSORT, a subcellular localization predication software to predict subcellular localization of 907 up-ubiquitinated proteins. WoLF PSORT is an updated version of PSORT/PSORT II for the prediction of eukaryotic sequences. Download FIG S3, JPG file, 0.03 MB. Copyright © 2021 Liu et al. 2021 Liu et al. https://creativecommons.org/licenses/by/4.0/ This content is distributed under the terms of the Creative Commons Attribution 4.0 International license.

NH_3_ may play fungistatic role of inhibiting conidial germination at two levels. First, NH_3_-inducing protein misfolding resulted in down-expression of proteins related to protein synthesis. As shown in Table 1, as many as 115 down-expressed proteins were related to protein synthesis. Among these, 29 ribosomal large subunit proteins and 21 ribosomal small subunit proteins were down-expressed (Fig. S4A), 53 proteins related to RNA processing, ribosome biogenesis, and RNA transport were also down-expressed (Fig. S4B), and 12 aminoacyl-tRNA synthases were down-expressed (Fig. S4C). Down-expression of these 115 proteins might have a negative impact on protein synthesis. Second, the UPR would be activated under ER stress. The PERK pathway of UPR can attenuate the protein synthesis by phosphorylation of eIF2a (49). In total, NH_3_ might repress the protein synthesis through these two methods. During conidial germination, the protein synthesis process needs to be initiated before RNA synthesis and DNA synthesis can begin (53). Our prior study showed that, in *A. oligospora*, the inhibition of protein synthesis resulted in inhibition of conidial germination (39). Thus, inhibition of protein synthesis should be a fungistatic mechanism of NH_3_ inhibiting conidial germination. This mechanism can be used to explain the antibiotic strategy of ammonia-producing *Streptomyces* species. The fungal diversity in soils may be regulated by microbial antagonisms that produce ammonia.

10.1128/mSystems.00879-21.7 FIG S4 Pathway analysis of down-expressed proteins related to protein synthesis. Besides ribosomal proteins (A), as is well known, protein translation depends on the participation of tRNA, rRNA, mRNA, and U-rich small nuclear RNA (Usn RNA). All these RNA molecules function after their transport from the nucleus to cytoplasm (Fig. S3B). However, before transportation, RNA molecules need to undergo a maturation process, for example, 5′-end processing, 5′-end capping, and 3′-end processing. Thereafter, RNA molecules are transported to the cytoplasm with the help of export proteins and nuclear pore complexes. UsnRNA matures in the cytoplasm, is then transported to the nucleus, and is involved in ribosome biogenesis. Pre-rRNA and ribosomal proteins are assembled as the ribosome large and small subunit through the ribosome biogenesis process. Moreover, during protein translation, the amino acids are ligated to tRNA and then transported to the ribosome for translation elongation. Twelve aminoacyl-tRNA synthases that catalyze the ligation were also down-expressed in fungistatic conidia (Fig. S3C). Download FIG S4, JPG file, 0.6 MB. Copyright © 2021 Liu et al. 2021 Liu et al. https://creativecommons.org/licenses/by/4.0/ This content is distributed under the terms of the Creative Commons Attribution 4.0 International license.

In addition, our knowledge of the mechanism of soil fungistasis will be beneficial for developing strategies to suppress soil pathogenic fungi through the manipulation of fungistasis. Fungistatic compounds are direct tools for soil microorganisms to achieve soil fungistasis. The mechanism of ammonia fungistasis might be common for other fungistatic compounds and soils. Based on the mechanism of ammonia fungistasis, new strategies can be explored to suppress the soil pathogenic fungi by replacing agricultural fungicides that would pollute the soil and water. For example, the endoplasmic reticulum-targeting compounds that induce ER stress (54) can be used to enhance the role of soil fungistasis and suppress the soil pathogenic fungi.

In addition, the survival strategy of conidia under ammonia inhibition provides a direction for developing methods that help biocontrol fungal agents overcome the inhibition of soil fungistasis and work effectively in soil. For example, nematophagous fungi are used to develop biocontrol agents; however, the soil fungistasis strongly inhibits their germination and hyphal growth. This inhibition may be relieved by maintaining ER homeostasis under the stress of soil fungistasis. We can reduce the ER stress induced by misfolded proteins by attenuating the protein synthesis rate, activating the ubiquitin-proteasome system, and regulating the metabolism to neutralize or degrade the fungistatic compounds by adding proper nutrients. Regulation of the UPR pathways is a key method for maintaining ER homeostasis, and BiP is a key target protein for regulating UPR.

## MATERIALS AND METHODS

### Testing the fungistatic role of ammonia.

To collect the conidia, *Arthrobotrys oligospora* ATCC 24927 was incubated on corn meal agar (CMA) plates at 28°C for 14 days. After that, the conidia were harvested and used for preparation of three conidial samples (fresh conidia, germinated conidia, and fungistatic conidia) according to a previously reported method (39). For the preparation of fungistatic conidia, the fresh conidia were resuspended in a suitable volume of sterile water to obtain a concentration of approximately 10^6^ CFU/ml. In one of the two-compartment petri dishes (diameter = 9 cm), 5 ml of 2% water agar (WA) was poured, and 50 μl of conidial suspension was spread on the WA surface. A tampon containing different volumes of ammonia water (wt/wt, 25% to 28%; Guanghua Sci-Tech Co., Ltd.) was placed in another compartment of the dish. The plates were then wrapped in Parafilm (Bemis, USA). All treatments were conducted in triplicate. After incubation at 20°C for 24 h, the conidial germination rate was determined under a microscope. Three microscope fields were observed for each plate. Conidia were considered germinated when the germ tube was observed.

To test the effect of alkaline pH on conidial germination, deionized water was adjusted to alkaline pH using 1 mM/liter sodium hydroxide. Then, 50 μl of conidial suspension was added to 5 ml deionized water with different pH, and the conidial germination rate was determined according to the above method. To test the inhibitory effect of ammonium ions on conidial germination, different volumes of ammonium chloride solution were added to 5 ml deionized water. The molar of NH_4_Cl in deionized water was same as that of NH_3_ in 7, 8, and 9 μl of ammonia water, respectively. Then, 50 μl of conidial suspension was added to the deionized water, and the conidial germination rate was determined according to the above protocol; 1 μl of ammonia water can provided maximumly about 0.22 mM/liter NH_3_ in the air contained in two-compartment petri dishes (diameter = 9 cm).

### Detection of ubiquitination by Western blotting and determination of ubiquitylome and transcriptome.

Three conidial samples, including fresh conidia, germinated conidia, and ammonia fungistatic conidia, were prepared according to a previously described method (39). Fresh conidia were harvested from fresh cultures and used as a control. To prepare germinated conidia, fresh conidia were allowed to germinate on CMA plates at 20°C for 24 h. The fungistatic conidia were generated according to the above-described method by spreading the fresh conidia on the CMA plate and incubating them for 24 h at 20°C under the fungistasis stress of 9 μl of ammonia water. In order to take the conidia from the agar medium, a layer of glass paper was placed on the medium surface.

Fresh, germinated, and fungistatic conidia (0.2 g) were used for protein extraction according to a previously described method (39). The total amounts of proteins were adjusted to the same amount by running short SDS-PAGE (0.3 cm), staining with Coomassie blue G-250, and quantifying the amounts of proteins using gray value quantification (Image-Pro Plus version 6.0; Media Cybernetics, USA) as described previously (55). According to the protein quantification results, the same amounts of proteins of the three samples were loaded for SDS-PAGE and Western blotting according to previously described methods (56), and the primary anti-ubiquitin antibody (ab19247; Abcam, China) and horseradish peroxidase-labeled secondary antibody (ab6721; Abcam) were used.

To analyze the transcriptome, at least 0.3 g of fresh, germinated, and fungistatic conidia, which have two duplicates, were sent to the Beijing Genomics Institute (BGI, China) for transcriptome sequencing using a reported method (57). The same amounts of three conidium samples with two duplicates were sent to PTM BIO Co., Ltd. (Hangzhou City, China) for quantitative ubiquitylome determination. Protein extraction and digestion, immune-affinity enrichment of the ubiquitinated peptides, and liquid chromatography tandem mass spectrometry (LC-MS/MS) analysis was performed following previously reported methods (58). The raw data were processed using the MaxQuant search engine (version 1.5.2.8) and searched against the target protein sequence database of *A. oligospora* ATCC 24927 from NCBI (reference sequence [RefSeq] no. NW_011645558.1). Trypsin/P was specified as a cleavage enzyme, allowing up to four missing cleavages. The mass tolerance for precursor ions was set as 20 ppm in the first search and 5 ppm in the main search, and the mass tolerance for fragment ions was set as 0.02 Da. Carbamidomethyl on Cys was specified as a fixed modification, and acetylation modification and oxidation on Met were specified as variable modifications. The false-discovery rate (FDR) was adjusted to <1%, and the minimum score for modified peptides was set at >40.

### Comparison of ubiquitination levels among samples with heat map.

For the ubiquitylome, the average ubiquitination values of each ubiquitinated site were calculated with the ubiquitination values from two duplicates. Then, the heat map was drawn with the average ubiquitination values using the R package pheatmap (version 2.0.3; https://cran.r-project.org/web/packages/cluster/).

### Ultrastructural analysis.

The ultrastructures of the fresh and fungistatic conidia were analyzed by transmission electron microscopy (JEM-1011; JEOL, Japan). The sample fixing, washing, dehydrating, saturating, embedding, and ultrathin slicing were performed according to the described methods (59).

### Correlation analysis of proteome, ubiquitylome, and transcriptome.

Using the ubiquitylome data of fresh conidia as the control, a ubiquitinated site with a quantitative ratio over 1.5-fold was considered upregulated. In contrast, a quantitative ratio below 1/1.5 was considered ubiquitination downregulated. For the transcriptome, the average fragments per kilobase per million (FPKM) values of genes in germinated and fungistatic conidia were calculated and compared to those in fresh conidia. A quantitative ratio of >2 or <0.5, was used for screening upregulated or downregulated genes. The proteome data were obtained from our previous study (39), in which a 2-fold selection criterion was used for screening differentially expressed proteins, and many proteins that did not reach the selection threshold were missed. In this study, in order to provide more information, a 1.5-fold selection threshold was used for selecting differentially expressed proteins.

The differentially ubiquitinated proteins and the differentially transcribed genes were compared to the differentially expressed proteins using a Venn diagram. The Venn diagram was drawn using the Web tool Venny version 2.1.0 (https://bioinfogp.cnb.csic.es). Moreover, these proteins or genes were annotated using the KEGG online service tool KAAS, and the annotation results were mapped on the KEGG pathway database using the KEGG online service tool KEGG Mapper. The KEGG pathway distribution of down-expressed proteins was regressively analyzed with that of differentially ubiquitinated proteins or differentially transcribed genes using the protein or gene number in KEGG pathways.

In order to distinguish the differentially regulated proteins or genes of different omics, the differentially regulated proteins from the proteome are named up-expressed or down-expressed proteins, the differentially regulated proteins from the ubiquitylome are named up-ubiquitinated or down-ubiquitinated proteins, and the differentially regulated genes from the transcriptome are named up-transcribed or down-transcribed genes.

### Testing the potential relief effects of nutrients on fungistatic stress of ammonia.

First, to prepare water agar (WA) media containing different nutrients, 3% glucose (g/100 ml, about 0.17 mol/liter), 0.17 mol/liter 2-oxoglutarate, 0.17 mol/liter pyruvate, and 0.17 mol/liter glutamate, respectively, were added to the WA medium. WA medium without the added nutrients was used as the control. These WA media were poured into one side of a two-compartment petri dish (diameter = 9 cm); every dish containing 5 ml WA medium and three duplicates were set. The conidial suspension was spread on the WA surface, and different volumes of ammonia water were placed on the other side of the dish according to the above-mentioned method for testing the fungistatic role of ammonia. The culture conditions and methods for determining the germination rate of conidia were the same as those described above. The potential relief effects of these nutrients on fungistatic stress of ammonia were assessed by comparing the conidial germination rates.

### Deletion of genes *AOL_s00054g963* and *AOL_s00210g337*.

The disruption vectors of genes *AOL_s00054g963* and *AOL_s00210g337* were constructed following a previously described procedure (39, 57). The primer pairs 963-5f/963-5r and 963-3f/963-3r were used for PCR amplification of the upstream and downstream homologous fragments of *AOL_s00054g963*, respectively. The primer pairs 337-5f/337-5r and 337-3f/337-3r were used to amplify the upstream and downstream homologous fragments of *AOL_s00210g337*, respectively. The primer pair hphF/hphR was used to amplify the hygromycin cassette from the vector pCSN44 (60). The upstream and downstream homologous fragments, the hygromycin cassette fragment, and the gapped yeast shuttle vector pRS426 (61) were cotransformed into the yeast strain FY834. The circular constructs were created by homologous recombination in yeast, and the disruption vectors (pRS426-g963 and pRS426-g337) were extracted from yeast and recovered by transformation into Escherichia coli DH5a.

Plasmids pRS426-g963 and pRS426-g337 were transformed into *A. oligospora* using a protoplast-based protocol (62, 63) and cultured on PDA medium supplied with molasses, saccharose, and 200 μg/ml hygromycin B (63). After transformation, the protoplasts were spread-plated on hygromycin-containing plates and cultured at 28°C for 7 days. Then, hygromycin-resistant transformants were selected and used to identify double crossover homologous recombinants by PCR. The primer pairs 963F/963R and 337F/337R were used to identify the double crossover mutants of genes *AOL_s00054g963* and *AOL_s00210g337*, respectively. The positive transformants were subcultured five times and confirmed by PCR again to select genetically stable transformants. The primers are listed in Text S1.

10.1128/mSystems.00879-21.8 TEXT S1 Sequence of primers used in the manuscript. Download Text S1, DOCX file, 0.02 MB. Copyright © 2021 Liu et al. 2021 Liu et al. https://creativecommons.org/licenses/by/4.0/ This content is distributed under the terms of the Creative Commons Attribution 4.0 International license.

### Functional evaluation of genes *AOL_s00054g963* and *AOL_s00210g337* in the survival of conidia under the inhibition of ammonia and soil.

The functions of genes *AOL_s00054g963* and *AOL_s00210g337* in the survival of conidia under ammonia inhibition was evaluated by comparing the conidial germination rate of mutant and wild-type strains. The conidial germination rates at 24 h under fungistatic stress of 4, 5, 6, 7, 8, 9, and 10 μl of ammonia water were tested according to the above-mentioned method. After this, to remove the ammonia, the two-compartment petri dishes containing 8, 9, and 10 μl of ammonia water was opened for 15 min in a clean bench, and the tampon containing ammonia water was removed. Then, the germination rates of conidia in these petri dishes were tested after ammonia removal for 24 h. The ability of mutant and wild-type strains to survive ammonia was assessed by comparing the 24-h conidial germination rates under fungistatic stress of ammonia and the restored germination rates of conidia after ammonia removal.

To test the germination rates of mutant and wild-type strains under the inhibition of soil, soil suspensions were prepared according to the reported method (57, 64). Three soil samples were collected from the Yunnan Province of China, and then soils and deionized water were added to a beaker at mass ratios of 2.5:1 and 1:1 and mixed well to produce three high-fungistatic soil suspensions and three medium-fungistatic soil suspensions. The fresh conidia of mutant and wild-type strains were placed in dialysis bags (300 kDa; Spectrum, USA), and the dialysis bags was then placed in the soil suspensions and cultured at 28°C; three conidia duplicates were set. After 24 h, a portion of the conidia were picked with a pipette and used to test germination rates. Meanwhile, the dialysis bags were moved from the soil suspensions to deionized water and cultured at 28°C for 24 h. Then the conidia were removed from the dialysis bags, and the germination rates were tested.

### Data availability.

The clean read data were deposited in the National Omics Data Encyclopedia (https://www.biosino.org/node/, no. OEP1908120455). Mass spectrometry raw data were deposited in MassIVE (https://massive.ucsd.edu, no. MSV000084740).
