# Supplementary material for: Fungistatic Mechanism of Ammonia against Nematode-Trapping Fungus Arthrobotrys oligospora, and Strategy for This Fungus To Survive Ammonia
Source: mSystems. 2021 Sep 14;6(5):e00879-21. doi: 10.1128/mSystems.00879-21 (PMC8547478; doi:10.1128/mSystems.00879-21)
Supplement: TABLE S1 [file msystems.00879-21-st001.docx]

Table S1 The annotation of protein edcoded by *AOL_s00210g337*

| Target gene name | Query cover | identity |
| --- | --- | --- |
| Blast the human BiP protein | | |
| *AOL_s00210g337* | 96% | 66.93% |
| *AOL_s00215g255* | 92% | 64.64% |
| Blast the yeast BiP protein | | |
| *AOL_s00210g337* | 93% | 73.98% |
| *AOL_s00215g255* | 88% | 62.21% |
