# Supplementary material for: Fungistatic Mechanism of Ammonia against Nematode-Trapping Fungus Arthrobotrys oligospora, and Strategy for This Fungus To Survive Ammonia
Source: mSystems. 2021 Sep 14;6(5):e00879-21. doi: 10.1128/mSystems.00879-21 (PMC8547478; doi:10.1128/mSystems.00879-21)
Supplement: TABLE S2 [file msystems.00879-21-st002.docx]

Table S2 Up-regulated molecular chaperones

| Gene name | KOG NO. | KOG description |  |
| --- | --- | --- | --- |
| AOL_s00081g272 | KOG3003 | Molecular chaperone of the GrpE family | |
| AOL_s00210g337 | KOG0100 | Molecular chaperones GRP78/BiP/KAR2, HSP70 superfamily | |
| AOL_s00193g46 | KOG0841 | Multifunctional chaperone (14-3-3 family) | |
| AOL_s00054g505 | KOG3478 | Prefoldin subunit 6, KE2 family | |
| AOL_s00076g650 | KOG3048 | Molecular chaperone Prefoldin, subunit 5 | |
| AOL_s00079g127 | KOG0714 | Molecular chaperone (DnaJ superfamily) | |
| AOL_s00054g963 | KOG0184 | 20S proteasome, regulatory subunit alpha type PSMA3/PRE10 | |
| AOL_s00054g527 | KOG4098 | Molecular chaperone Prefoldin, subunit 2 | |
| AOL_s00083g458 | KOG3313 | Molecular chaperone Prefoldin, subunit 3 | |
| AOL_s00112g82 | KOG3470 | Beta-tubulin folding cofactor A | |
| AOL_s00215g289 | KOG0356 | Mitochondrial chaperonin, Cpn60/Hsp60p | |
| AOL_s00078g192 | KOG3192 | Mitochondrial J-type chaperone | |
| AOL_s00054g77 | KOG0102 | Molecular chaperones mortalin/PBP74/GRP75, HSP70 superfamily | |
| AOL_s00215g255 | KOG0101 | Molecular chaperones HSP70/HSC70, HSP70 superfamily | |
| AOL_s00075g169 | KOG0546 | HSP90 co-chaperone CPR7/Cyclophilin | |
| AOL_s00006g437 | KOG1641 | Mitochondrial chaperonin | |
| AOL_s00097g398 | KOG1760 | Molecular chaperone Prefoldin, subunit 4 | |
