# Supplementary figures and images for: Fungistatic Mechanism of Ammonia against Nematode-Trapping Fungus Arthrobotrys oligospora, and Strategy for This Fungus To Survive Ammonia
Source: mSystems. 2021 Sep 14;6(5):e00879-21. doi: 10.1128/mSystems.00879-21 (PMC8547478; doi:10.1128/mSystems.00879-21)

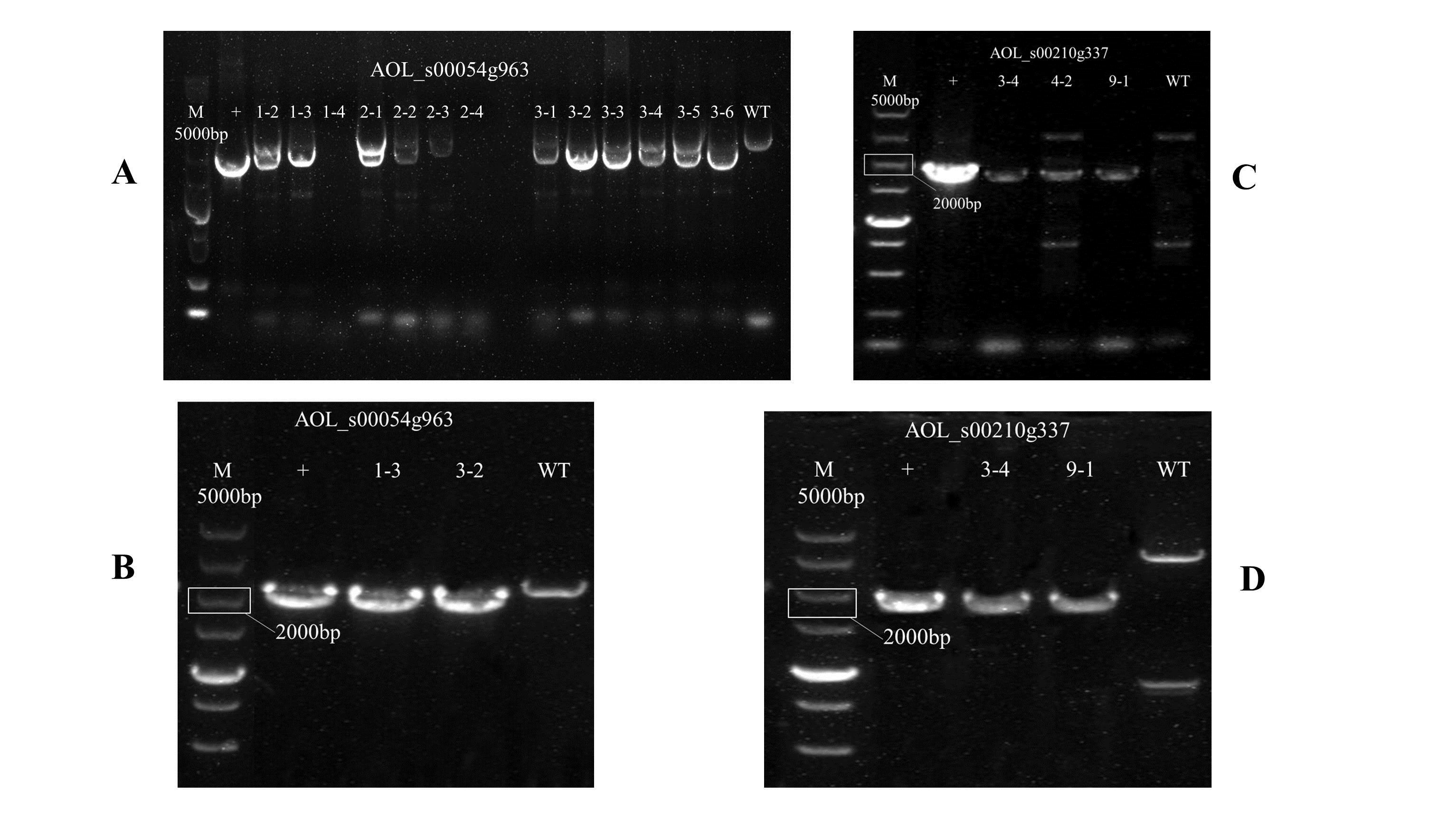

Supplement: FIG S1 [file msystems.00879-21-sf001.jpg]

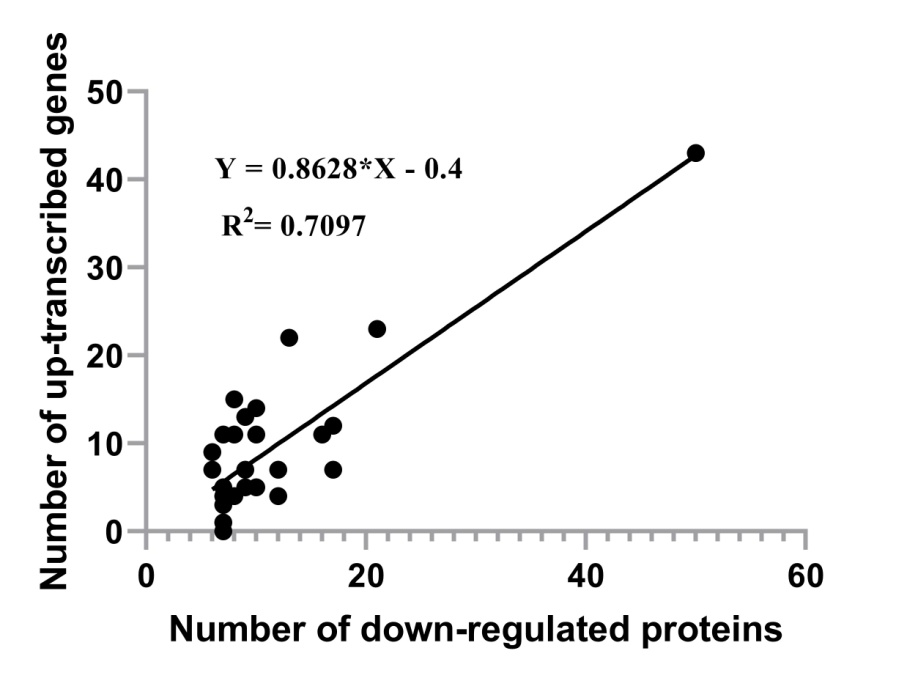

Supplement: FIG S2 [file msystems.00879-21-sf002.jpg]

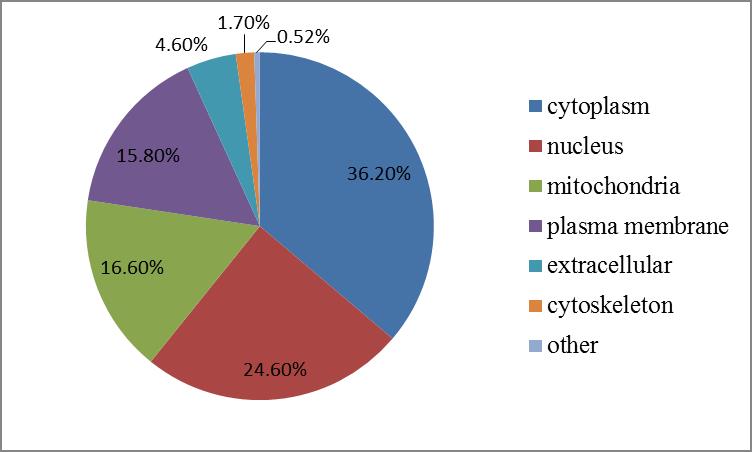

Supplement: FIG S3 [file msystems.00879-21-sf003.jpg]

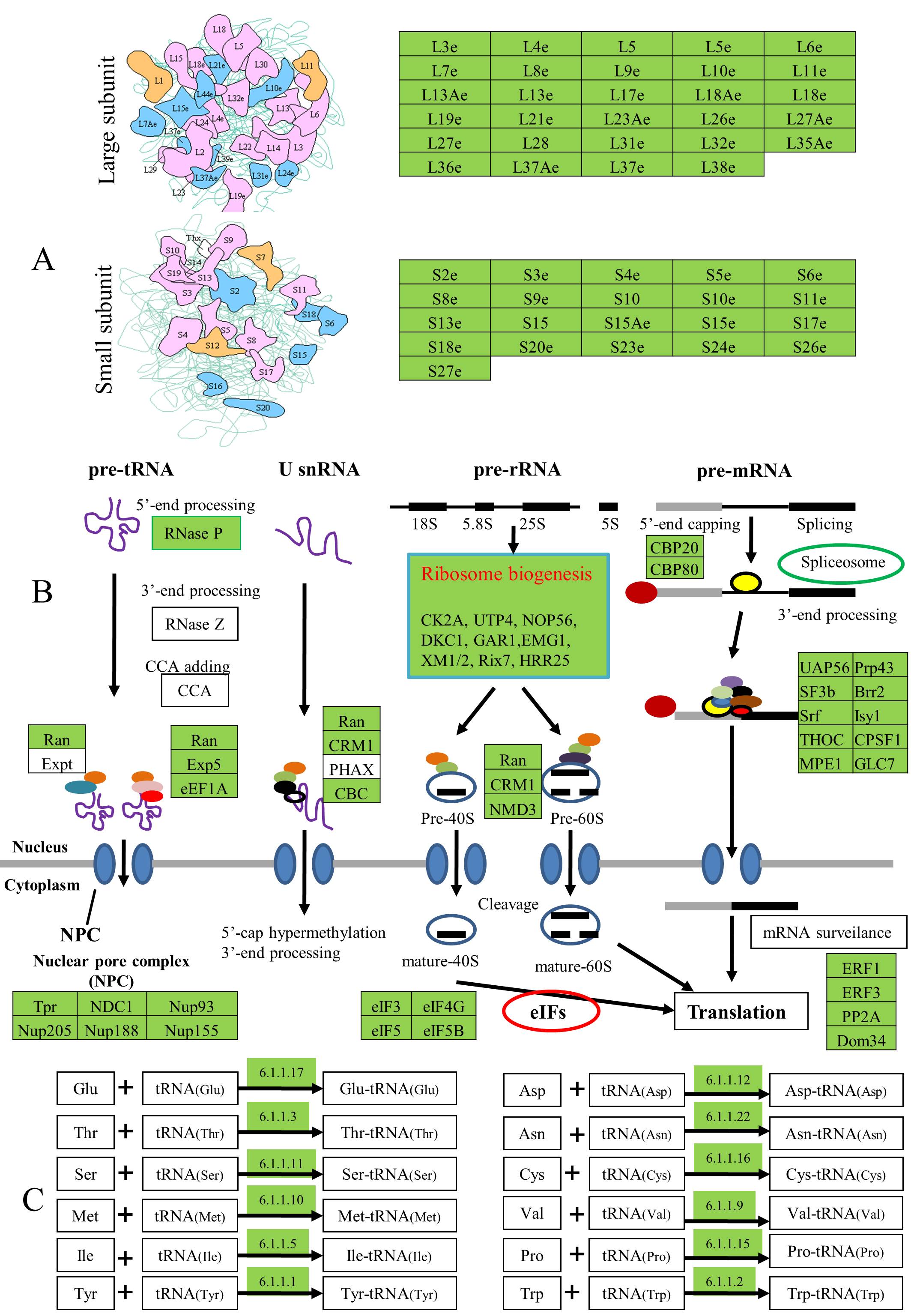

Supplement: FIG S4 [file msystems.00879-21-sf004.jpg]
