# Supplementary material for: Fungistatic Mechanism of Ammonia against Nematode-Trapping Fungus Arthrobotrys oligospora, and Strategy for This Fungus To Survive Ammonia
Source: mSystems. 2021 Sep 14;6(5):e00879-21. doi: 10.1128/mSystems.00879-21 (PMC8547478; doi:10.1128/mSystems.00879-21)
Supplement: TABLE S3 [file msystems.00879-21-st003.docx]

Table S3 Comparison of pathway types of 651 down expressed proteins and 1350 up transcribed genes

| KEGG pathways | Number of down expressed proteins | Number of up transcribed genes |
| --- | --- | --- |
| Ribosome | 50 | 43 |
| RNA transport | 21 | 23 |
| Cell cycle - yeast | 17 | 12 |
| MAPK signaling pathway - yeast | 17 | 7 |
| Meiosis - yeast | 16 | 11 |
| Ribosome biogenesis in eukaryotes | 13 | 22 |
| Ubiquitin mediated proteolysis | 12 | 7 |
| Aminoacyl-tRNA biosynthesis | 12 | 4 |
| Autophagy - yeast | 10 | 5 |
| Endocytosis | 10 | 14 |
| Alanine, aspartate and glutamate metabolism | 10 | 5 |
| mRNA surveillance pathway | 10 | 11 |
| DNA replication | 9 | 5 |
| RNA degradation | 9 | 7 |
| Spliceosome | 9 | 13 |
| Thermogenesis | 8 | 11 |
| Mitophagy - yeast | 8 | 4 |
| Protein processing in endoplasmic reticulum | 8 | 15 |
| mTOR signaling pathway | 8 | 4 |
| PI3K-Akt signaling pathway | 8 | 4 |
| Phospholipase D signaling pathway | 7 | 0 |
| Rap1 signaling pathway | 7 | 1 |
| Purine metabolism | 7 | 11 |
| Glycerophospholipid metabolism | 7 | 5 |
| Regulation of actin cytoskeleton | 7 | 3 |
| Pyruvate metabolism | 7 | 4 |
| Sphingolipid signaling pathway | 7 | 3 |
| Insulin signaling pathway | 7 | 4 |
| Ras signaling pathway | 7 | 3 |
| Starch and sucrose metabolism | 6 | 9 |
| 2-Oxocarboxylic acid metabolism | 6 | 7 |
| Amino sugar and nucleotide sugar metabolism | 6 | 7 |
