# Supplementary material for: Fungistatic Mechanism of Ammonia against Nematode-Trapping Fungus Arthrobotrys oligospora, and Strategy for This Fungus To Survive Ammonia
Source: mSystems. 2021 Sep 14;6(5):e00879-21. doi: 10.1128/mSystems.00879-21 (PMC8547478; doi:10.1128/mSystems.00879-21)
Supplement: TEXT S1 [file msystems.00879-21-s0001.docx]

Primer sequence

963-5f :

5’-GTAACGCCAGGGTTTTCCCAGTCACGACGGCGGATAGATAACCCCCATT-3’ 963-5r:

5’-ATCCACTTAACGTTACTGAAATCTCCAACTTCGGAGCGTGGTATTTTG-3’

963-3f :

5’- CTCCTTCAATATCATCTTCTGTCTCCGACGAGGGAGCAAGTAAGTAAGAAGC -3’

963-3r:

5’- GCGGATAACAATTTCACACAGGAAACAGCCGCAAGTTAGCAGGATGAAG -3’

337-5f :

5’- GTAACGCCAGGGTTTTCCCAGTCACGACGACCACTCGCTCCCACCTCTA -3’ ;

337-5r:

5’- ATCCACTTAACGTTACTGAAATCTCCAACAGCAGCCCAATCAGACACG -3’

337-3f :

5’- CTCCTTCAATATCATCTTCTGTCTCCGACGTCTAAACACGGAGAACAGGC -3’ ;

337-3r:

5’- GCGGATAACAATTTCACACAGGAAACAGCAGCATAACGAAAGTCTGGGG -3’)

hphF : 5’- GTTGGAGATTTCAGTAACGTTAAGTGGAT -3’

hphR: 5’- GTCGGAGACAGAAGATGATATTGAAGGAGC -3’

963F : 5’- GGTTTTCGGGTTCTTGGTCTC -3’

963R : 5’- CGGCTATGCTACGATTATGGG -3’

337F : 5’- GAAAAATAGGCTGTGAAGAAGGC -3’

337R : 5’- CTATCTTGTGGTTTTGTTGTGGC -3’
